# Early postoperative HPA-axis testing after pituitary tumor surgery: reliability and safety of basal cortisol and CRH test

**DOI:** 10.1007/s12020-019-02094-6

**Published:** 2019-09-25

**Authors:** Friso de Vries, Daniel J. Lobatto, Leontine E. H. Bakker, Wouter R. van Furth, Nienke R. Biermasz, Alberto M. Pereira

**Affiliations:** 1 Department of Medicine, Division of Endocrinology, Leiden University Medical Centre, Leiden, The Netherlands; 2 Center for Endocrine Tumors Leiden (CETL), Center for Pituitary Care, Leiden University Medical Center, Leiden, The Netherlands; 3 Department of Neurosurgery, Leiden University Medical Centre, Leiden, The Netherlands

**Keywords:** Adrenal insufficiency, HPA-axis, Cortisol, Transsphenoidal surgery, Pituitary, Postoperative testing

## Abstract

**Purpose:**

To assess the reliability and safety of a postsurgical evaluation strategy of adrenal function using CRH stimulation and basal cortisol concentrations after transsphenoidal pituitary surgery.

**Methods:**

Retrospective cohort study of all patients undergoing endoscopic transsphenoidal surgery from 2010 to 2017, in whom early postoperative basal cortisol and/or CRH-stimulated cortisol secretion were available, including confirmation of adrenal function during follow-up. Patients with Cushing’s disease were excluded. Optimal test performances were assessed using ROC analysis.

**Results:**

A total of 156 patients were included. Sensitivity and specificity of the CRH test were 78% and 90%, respectively, and 86% and 92% for basal cortisol, respectively, using an optimal cutoff of 220 nmol/L. Eight patients had false-negative test results with the CRH test (normal test but adrenal insufficient at follow-up), and six patients with basal cortisol, the majority of which had multiple pituitary hormone deficiencies and fluid imbalances. No clinical adverse events occurred in patients with false-negative test results. The diagnostic performance of a single basal cortisol measurement was superior to the CRH test.

**Conclusions:**

The early postoperative basal cortisol is a safe and simple measurement to guide (dis)continuation of hydrocortisone replacement. However, disturbing factors, e.g., sodium balance disorders, contraceptives, untreated hypopituitarism, and illness impact the interpretation and in those cases this measure is unreliable. We propose an algorithm in which hydrocortisone replacement at discharge is based on basal cortisol <220 nmol/L on postoperative day 2 or 3 in a stable condition.

## Introduction

Transsphenoidal endoscopic surgery is the cornerstone of treatment for the majority of patients with pituitary tumors. A possible complication of surgery is the onset of new pituitary insufficiencies. This may eventually recover in some, but not in all patients. Correct interpretation of adrenal function early after surgery is of paramount importance. Cortisol deficiency may be life-threatening and unnecessary glucocorticoid replacement can be harmful and may cause (long-lasting) side effects. A meticulous postsurgical assessment of adrenal function is therefore mandatory. However, there is no consensus on how to evaluate adrenal function directly after surgery.

Several tests are available for the assessment of adrenal function, of which the insulin tolerance test (ITT) is considered as the gold standard. The ITT, however, is not suitable for the immediate postoperative period, as it is burdensome, and has contraindications. The ACTH test may not detect cases of new-onset secondary adrenal insufficiency (AI). Alternatives are the CRH-stimulation test, the metyrapone test, and measurements of nonstimulated basal cortisol, or random serum cortisol concentrations [1–5]. Available data assessing the safety and accuracy of these tests are limited [2, 3, 6–12]. Moreover, a comparison between all different assessment methods in the postoperative setting has not yet been performed.

The Center for Endocrine Tumors Leiden is a tertiary referral center and coordinating center of the European Reference Network for Rare Endocrine Conditions (Endo-ERN, www.endo-ern.eu). At our Reference Centre for pituitary care, a CRH-stimulation test ~5 days after surgery has been the preferred test to evaluate adrenal function after surgery since 1990. We previously reported on the clinical applicability and safety of the test in our cohort of patients treated between 1990 and 2009. This study stated that the strategy to continue hydrocortisone replacement guided by the CRH test appeared to be safe and did not result in any case of adrenal crises [4]. Although safe, the early postoperative CRH test could not reliably predict long-term adrenal function. Therefore, retesting was considered mandatory. Nowadays, the introduction of endoscopic surgery and lower perioperative hydrocortisone replacement doses allow earlier discharge in a significant proportion of patients. This prompted us to critically re-evaluate our postsurgical evaluation strategy of adrenal function.

The aim of the present study was to re-assess the current practice by studying performance and clinical safety of the CRH test directly after surgery in diagnosing AI as compared with a confirmation test during follow-up. Next, we assessed the reliability and safety of a simplified protocol based on a postoperative single basal cortisol measurement only. Performances of both tests were compared in order to optimize the postsurgical evaluation of adrenal function.

## Methods

### Patient selection

**Fig. 1:**
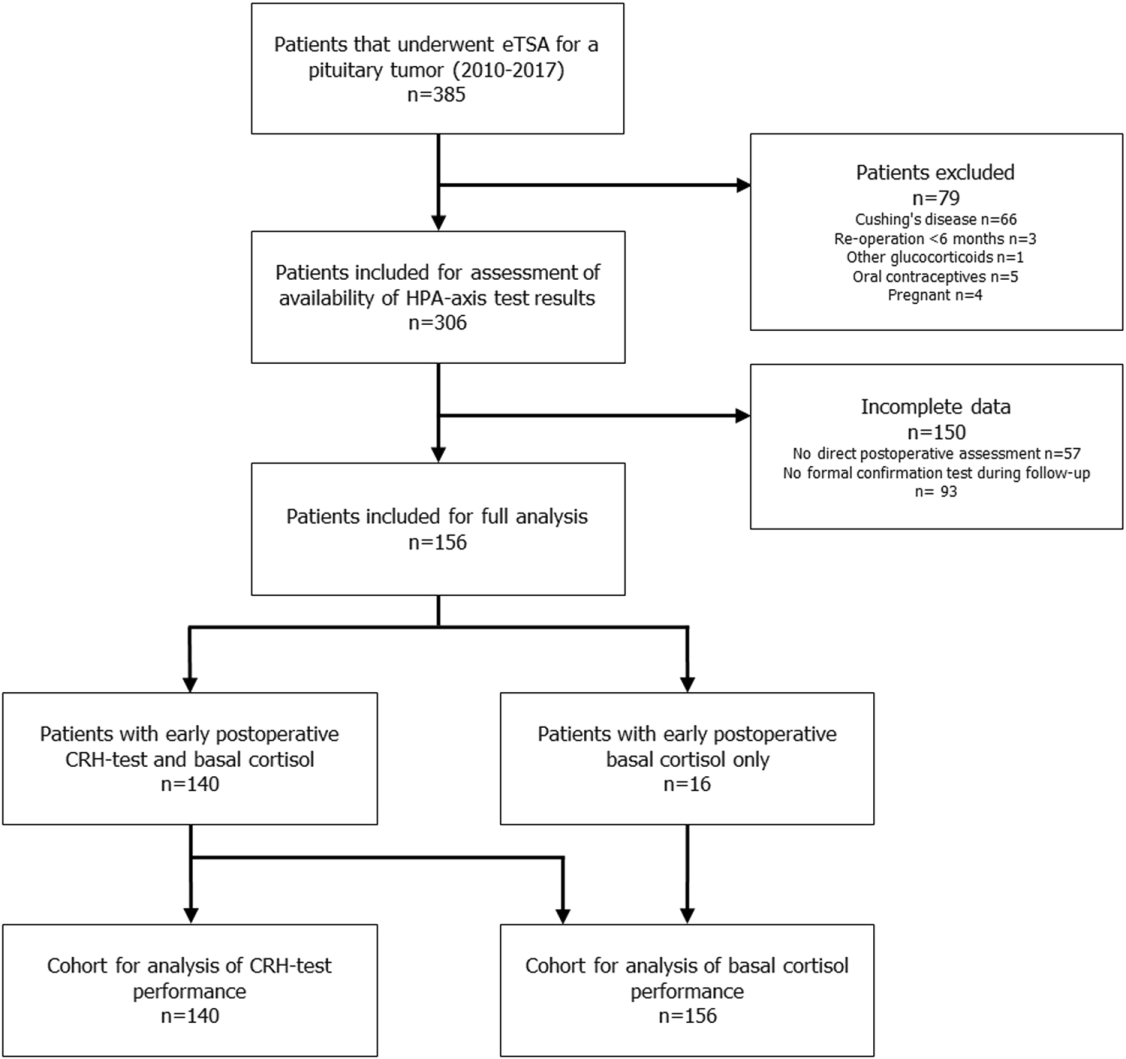
Flowchart of patient selection

(Fig. 1) We performed a retrospective chart review of all consecutive patients who underwent endoscopic transsphenoidal pituitary surgery at our center between January 2010 and December 2017 (*n* = 385). Patients were excluded in case of Cushing’s disease (*n* = 66), re-operation within 6 months (*n* = 3), treatment with glucocorticoids other than hydrocortisone (*n* = 1), use of oral contraceptives (*n* = 5), and pregnancy (*n* = 4). Patients with available data on early postoperative testing (basal cortisol concentrations and/or CRH test) and a conclusive confirmation test during follow-up were eligible for inclusion. Early postoperative testing was defined as a maximum of 2 weeks after surgery. A conclusive confirmation test was defined as a cortisol response after stimulation with either CRH or ITT or a basal cortisol below or above the reference range. Basal cortisol values within the reference ranges in the absence of a stimulation test were considered uninterpretable and these patients were excluded. In 57 patients, no direct postoperative evaluation of residual cortisol secretion was performed and in 93 patients no formal confirmation test was performed during follow-up. This resulted in the inclusion of 156 patients (40.5% of the full cohort). In 16 patients (10.3%) results of early basal cortisol were available, but not of an early CRH test.

### Management regarding hydrocortisone replacement

On the day of surgery, patients received 50 mg/24 h intravenous hydrocortisone continuously. On postoperative day 1 this was switched to a 20-10-10 mg oral dose, and from postoperative day 2 onwards 10-5-5 mg hydrocortisone was administered. Five days after surgery, or later depending on the clinical situation, a CRH test was performed. Hydrocortisone was withdrawn prior to discharge in case of a normal cortisol response. Hydrocortisone was continued in case of an abnormal peak cortisol at least until the patient was retested (usually within the first 6 months). Patients with very low cortisol levels and evident hypopituitarism were not retested, patients with high cortisol responses after CRH-stimulation and no other signs of hypopituitarism were not routinely retested, based on the clinicians’ decision. All patients were closely monitored and adrenal function was re-evaluated in case of clinical suspicion of possible AI.

### Endocrine assessment

#### CRH test

Patients were instructed to take the last hydrocortisone dose at least 18 h prior to the test. After an overnight fast, 100 µg of corticoliberine (human CRH) (Ferring Pharmaceuticals, Hoofddorp, the Netherlands) was administered intravenously, and blood samples were collected for measurement of cortisol and ACTH at −15, −5, 15, 30, 45, and 60 min after CRH administration. Peak cortisol of >430 nmol and a peak ACTH of >40 ng/L indicated a normal response to CRH stimulation [13, 14].

#### Basal cortisol level

Blood samples were taken between 6 and 8 A.M. after withdrawing hydrocortisone for at least 18 h. Cortisol concentrations <70 nmol/L were considered as evidence of AI. Cortisol concentrations of >430 nmol/L as evidence of normal HPA-axis functioning. Intermediate values were regarded as inconclusive and indicated further (dynamic) testing.

#### ITT

The ITT was performed after an overnight fast after withdrawal of hydrocortisone replacement for at least 18 h. A total of 0.1 U/kg body weight of intravenous insulin (Novorapid, Novo Nordisk Farma, Bagsvaerd, Denmark) was administered to induce adequate hypoglycemia (defined as nadir glucose <2.2 mmol/L in the presence of neuroglycopenic symptoms). Blood samples were drawn for the measurement of cortisol, ACTH, and GH at −15, −5, 15, 45, 60, and 90 min after insulin administration. Peak cortisol of >430 nmol/L was defined as a normal response to hypoglycemia.

#### ACTH test

The ACTH test was performed after an overnight fast after withdrawal of hydrocortisone replacement for at least 18 h. Tetracosactide 250 µg (Synacthen, Novartis, Arnhem, the Netherlands) was administered and blood is drawn for cortisol and ACTH levels at −15, −5, and 30 min after injection of ACTH. A normal cortisol response was defined as peak cortisol >430 nmol/L. An ACTH test was deemed reliable after at least 12 months after surgery.

Adrenal crises were defined and graded according to the expert opinion paper by B. Allolio [15].

### Assays

Cortisol was measured using electrochemiluminescent immunoassay (ECLIA) on a Cobas 8000 module e602 (Roche Diagnostics, Mannheim, Germany). Up to January 1st, 2016, blood samples were first treated with Elecsys Cortisol I reagent. From January 1st, 2016 onward, the Elecsys Cortisol II reagent was used. The use of the cortisol II reagent results in a factor 0.78 lower cortisol values than compared with the cortisol I reagent. We transformed each result of the cortisol I reagent to a cortisol II result (multiplied by 0.78). ACTH was measured using ECLIA on the same Cobas module and with an ACTH reagent (Roche Diagnostics, Mannheim, Germany). The detection limit ranges from 1 to 2000 ng/L.

### Statistical analysis

Statistical analysis was performed using IBM SPSS statistics 23 (SPSS Inc. Chicago, Illinois, USA). Descriptive statistics were used for baseline characteristics, with continuous variables being reported as means with range. Separate analyses were performed for the performances of the CRH test and basal cortisol. Contingency tables were used to calculate the performance indices of both tests. The positive likelihood ratio (LR+) was defined as sensitivity divided by 1-specificity. Inversely, the negative LR− was calculated as 1-sensitivity divided by specificity. Receiver operator characteristics (ROC) were performed for determining optimum cut-off values. Youden’s index (sensitivity + specificity-1) was used to assess the optimal cutoff. First, the results of early postoperative testing were compared with the result of an ITT during follow-up (gold standard). Because of the low sample size, thereafter the results of early postoperative testing were compared with all confirmation tests during follow-up. The obtained cut-off values were used to assess the diagnostic performance of the tests. Because the obtained cut-off values in this study highly adhere to the cohort and underdiagnosis of AI can be dangerous, we chose cut-off values that are somewhat on the safe side.

## Results

### Baseline characteristics

One hundred and fifty-six patients were included, with the following diagnoses: pituitary adenoma (Cushing’s disease excluded): *n* = 140 (89.7%), craniopharyngioma: *n* = 9 (5.8%), and Rathke’s cleft cyst: *n* = 7 (4.5%) (Table 1). Mean age was 53.2 years, and 51% of patients were female. Thirty-four patients (21.8%) were adrenal insufficient before surgery. Mean follow-up was 4.2 years (range 0.74–8.64 years), with a total of 656 patient-years of follow-up.

**Table 1:** Baseline characteristics

Baseline characteristics	Number of patients (*n* = 156)
M/F	76/80
Mean age (years)	53.2 (range 17–85)
Diagnosis	
Non-functioning adenoma	98
GH-producing adenoma	22
Prolactinoma	20
Other adenoma	7
Craniopharyngioma	11
RCC	8
Preoperative pituitary function	
No deficiencies	80
Preoperative single pituitary deficiency	26
Preoperative multiple pituitary deficiencies	48
Preoperative AI	34
Not documented	2^a^
Confirmation test^b^	
Single cortisol measurement	39
ITT	63
CRH test	52
ACTH test	2

^a^In two patients no preoperative hormonal panel was performed because of immediate surgery following pituitary apoplexy

^b^During follow-up (mean 8.0 months after surgery)

### ROC analysis of early postoperative CRH test

**Fig. 2:**
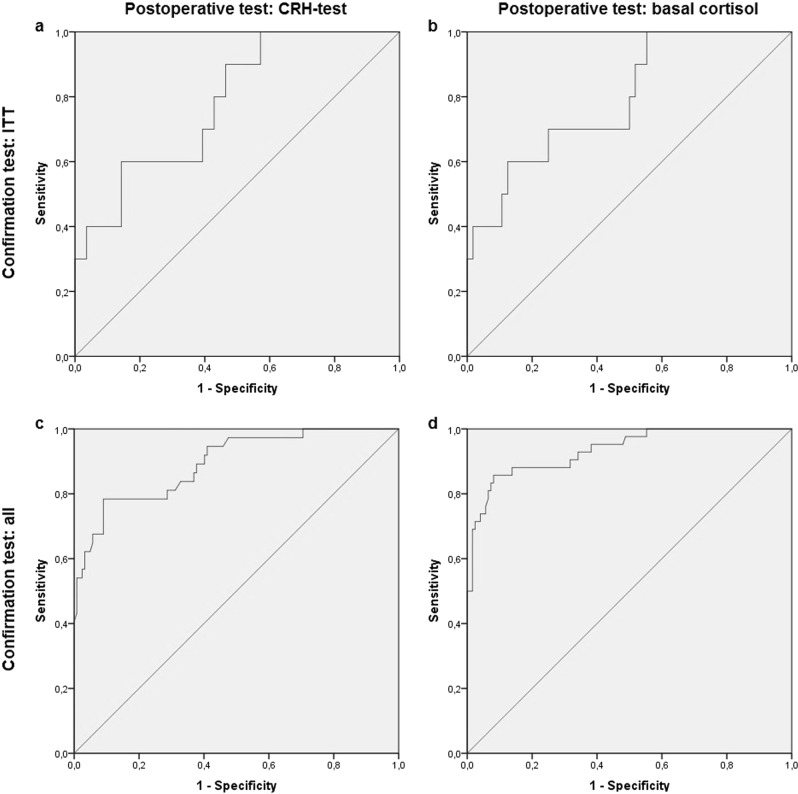
ROC-curve of CRH test and basal cortisol as confirmed with ITT and all confirmation tests, with reference line (diagonal). ROC-curve of CRH test and basal cortisol as confirmed with ITT and all confirmation tests, with reference line (diagonal). **a** CRH test vs ITT, AUC: 0.767 (95%CI 0.614–0.920), **b** Basal cortisol vs ITT, AUC: 0.767 (95%CI 0.608–0.927), **c** CRH test vs all confirmation tests, AUC: 0.885 (95%CI 0.817–953), **d** Basal cortisol vs all confirmation tests, AUC: 0.928 (95%CI 0.879–976)

Results of early postoperative CRH testing with subsequent ITT during follow-up were available in 61 patients (Fig. 2a, c). The ROC analysis of the early postoperative CRH test with the ITT as reference showed an AUC of 0.767 (95%CI 0.614–0.920). A peak cortisol level of 574 nmol/L corresponded with 100% sensitivity, and a specificity of 46%, whereas a cutoff of 310 nmol/L corresponded to a specificity of 100%, but with only 22% sensitivity. The optimum cut-off value was 424 nmol/L, with a Youden index of 0.421 (sensitivity 55.6% and specificity 86.5%).

A ROC analysis was also performed for all different confirmation tests (*n* = 140) which yielded nearly the same optimal cutoff with higher statistical performances: 430 nmol/L. The upper cutoff in this analysis was 672 nmol/L and the lower cutoff 232 nmol/L. Therefore, a CRH-stimulated cortisol concentration of 430 nmol/L (the already used cutoff in daily practice) was used as the cutoff for test performance. Performance indices for both references are presented in Table 4.

### Early postoperative CRH testing versus confirmation test during follow-up (*n* = 140 patients)

Early postoperative CRH testing showed a mean peak cortisol concentration of 545 nmol/L (range 10–1211).

A cortisol peak of below 430 nmol/L was observed in 32 patients (22.9%), all of whom continued hydrocortisone replacement at discharge. AI was confirmed in 23 of 32 patients (71.9%) at follow-up (ITT *n* = 12, CRH test *n* = 12, ACTH test *n* = 2, basal cortisol *n* = 6). Hydrocortisone was discontinued in nine patients with normal test results during follow-up (Table 2: patients 1–9).

**Table 2:** Patients with initial test results indicating adrenal insufficiency with normal adrenal function during follow-up

No.	Sex	Age (years)	Diagnosis	BC (nmol/L)	CRH-peak cortisol (nmol/L)	HC after surgery (y/n)	Confirmation test	Peak cortisol (nmol/L)	Other pituitary hormone deficiency	Follow-up	Clinical event
1	V	45	PRL-oma	278	313	If necessary	ITT	477	None	No complaints when not using HC. 3 borderline low CRH-tests. Adequate response to ITT.	N
2	M	45	GhA	373	378	Y	ITT	569	None	Adequate response to CRH test during follow-up. ITT thereafter adequate.	N
3	V	30	PRL-oma	269	384	If necessary	CRH	516	None	Did not use HC after CRH test. BC directly postoperative and at 3 months high intermediate. CRH test at 3 months sufficient.	N
4	V	42	Gh/PRL-oma	220	385	If necessary	ITT	438	Primary hypothyroidism	ITT 3 months postoperative adequate.	N
5	V	17	PRL-oma	424	391	If necessary	ITT	677	None	Felt good without HC, ITT 1 month postoperative adequate.	N
6	M	57	PRL-oma	342	427	If necessary	ITT	474	None	Borderline low CRH test. Did not use HC. Received radiotherapy 2 months postoperative. ITT 4 months postoperative showed adequate rise.	N
7	M	71	NFA	88	236	Y	CRH	446	None	Preoperative panhypopituitarism. During follow-up restoration of all hormonal axes. Adequate CRH test 5 months postoperative.	N
8	V	55	NFA	86	360	Y	ITT	573	GHD	Complaints of HC. Two insufficient CRH-tests, but felt better without HC. ITT 6 months postoperative sufficient.	N
9	M	47	NFA	193	362	Y	ITT	441	None	Forgot HC regularly without complaints. ITT 6 months postoperative sufficient.	N
10	V	23	PRL-oma	172	missing	If necessary	CRH	489	None	Felt good when not using HC. Two high intermediate random cortisol. CRH test 4 months postoperative sufficient.	N
11	V	33	PRL-oma	160	434	Y	ITT	522	None	Borderline adequate CRH test. Persistent complaints of fatigue, started taking HC. ITT 7 months postoperative adequate.	N
12	V	44	NFA	155	435	N	ITT	430	GHD, TSH	Borderline normal CRH test. No HC. 2 months postoperative ER presentation with hypotension. Start HC. ITT 4 months postoperative showed adequate rise.	N
13	V	61	NFA	199	437	N	CRH	441	TSH	Borderline adequate CRH test. CRH test 2 months later adequate.	N
14	V	43	NFA	212	441	N	ITT	514	None	Adequate CRH test. ITT 4 months postoperative showed sufficient response.	N
15	M	42	NFA	178	527	N	ITT	580	None	Adequate CRH test. ITT 6 months postoperative showed sufficient response.	N
16	V	33	NFA	147	753	N	ITT	599	None	Adequate CRH test. ITT 31 months postoperative showed sufficient response.	N

*BC* basal cortisol, *CRH* corticotropic releasing hormone-test, *HC* hydrocortisone, *ITT* insulin tolerance test, *PRL-oma* prolactinoma, *GhA* somatotroph adenoma, *NFA* nonfunctioning adenoma, *GH/PRL* growth hormone/prolactin co-secreting adenoma

The cortisol response to CRH early after surgery was above 430 nmol/L in 108 patients (77.1%), who discontinued hydrocortisone thereafter. Eight of these (7.4%) were diagnosed with AI during follow-up (ITT *n* = 4, CRH test *n* = 2, and basal cortisol *n* = 2, see Table 3: patients 1–8). Normal adrenal function was confirmed during follow-up in 100 patients, (ITT *n* = 45), CRH test *n* = 35, and basal cortisol *n* = 20). As can be appreciated from Table 3, two of these eight patients with a false-negative test result had new-onset hypothyroidism after surgery, and five patients had a concomitant diagnosis of DI or SIADH at the time of testing (*n* = 4) or which manifested early after the test (*n* = 1).

**Table 3:** Patients with initial test results indicating normal adrenal function, but with adrenal insufficiency during follow-up

No.	Sex	Age (years)	Diagnosis	Basal cortisol (nmol/L)	CRH-peak cortisol (nmol/L)	HC after surgery (y/n)	Confirmation test	Peak cortisol (nmol/L)	Other pituitary hormone deficiency	Follow-up	Clinical event
1	M	45	NFA	243	497	N	ITT	424	Panhypopit	Preoperative panhypopituitarism, adequate CRH test postoperative, but borderline cortisol response during ITT 4 months later with complaints of fatigue.	N
2	M	55	NFA	283	502	N	ITT	328	None	During follow-up insufficient ITT twice, however, never used HC.	N
3	M	65	NFA	318	525	N	ITT	381	GHD	Complaints of fatigue at 6 month follow-up with insufficient ITT. Complaints subsided after start HC.	N
4	M	44	Cranio	299	562	N	ITT	15	Panhypopit	Preoperative panhypopituitarism. DI with irregular desmopressin dose at the time of postoperative testing. ITT at 6 months follow-up very low. Four years later Addisonian crisis after vomiting out HC.	Y
5	M	60	NFA	363	528	N	CRH	361	TSH, LH/FSH	SIADH with fluid restriction on day of test (nadir sodium 129 mmol/L). At 6 month follow-up complaints of fatigue. Inadequate response after CRH-stimulation. Complaints subsided after start HC.	N
6	M	33	NFA	404	662	N	BC	48	TSH, LH/FSH	Hypothyroidism on day of test (fT4: 11.3 pmol/L). ER presentation with hyponatremia (126 mmol/L) and low cortisol (48 nmol/L) due to adrenal insufficiency 4 days after CRH test, HC restarted, adrenal insufficiency confirmed with basal cortisol at follow-up at 6 months.	N
7	V	80	NFA	84^a^	534	N	BC	11	TSH	Low urine production on day of test. Discharged with fluid restriction. Readmission 2 days later with hyponatremia (128 mmol/L) New secondary hypothyroidism. After start of LT4 complaints of fatigue and nausea, with very low BC. Start HC.	N
8	M	59	NFA	209^a^	539	N	CRH	51	TSH, LH/FSH	SIADH with urine production of 800 mL on day of test. New-onset secondary hypothyroidism. 2 months postoperative myalgia and fatigue. Complaints subsided after start HC and LT4. Both basal and CRH-stimulated cortisol very low.	N

*CRH* corticotropic releasing hormone-test, *HC* hydrocortisone, *ITT* insulin tolerance test, *BC* basal cortisol, *NFA* nonfunctioning adenoma, *Cranio* craniopharyngioma, *GHD* growth hormone deficiency

^a^Patients 7 and 8 had sufficient rise during the direct postoperative CRH test, but a basal cortisol below proposed cut-off value

### ROC analysis of basal (nonstimulated) cortisol

Results of early postoperative basal cortisol measures with subsequent ITT during follow-up were available in 63 patients (Fig. 2b, d). The ROC analysis of basal cortisol with the ITT as reference showed an AUC of 0.767 (95%CI 0.608–0.927). A basal cortisol concentration of 325 nmol/L corresponded with 100% sensitivity and a specificity of 42.6%. A cortisol cut-off of 82 nmol/L corresponded with 100% specificity and a sensitivity of 22.2%. The optimal cortisol cut-off value was 218 nmol/L or 325 nmol/L, with a Youden-index of 0.426 (sensitivity 55.6% and specificity 87.0%, and sensitivity 100% and specificity 42.6%, respectively).

A ROC analysis with all different confirmation tests (*n* = 156) as reference yielded nearly the same cutoffs with higher statistical performances, the higher cutoff in this analysis being 411 nmol/L, the lower 86 nmol/L, and the optimal cutoff 218 nmol/L, respectively.

Therefore, a cortisol concentration of 80 nmol/L (circa 86) was taken as the lower cutoff, 325 nmol/L as the higher cutoff, and 220 nmol/L as the optimal cut-off value. Performance indices for both references are presented in Table 4.

**Table 4:** Diagnostic performance indices of the early postoperative CRH stimulation test and of a single morning cortisol measurement

Test	CRH test >430 nmol/L vs ITT	Basal cortisol >220 nmol/L vs ITT	CRH test >430 nmol/L vs all tests	Basal cortisol >220 nmol/L vs all tests
Sensitivity	0.556	0.556	0.742	0.842
Specificity	0.865	0.870	0.917	0.916
Positive predictive value	0.417	0.417	0.719	0.762
Negative predictive value	0.918	0.922	0.926	0.947
Positive likelihood ratio	4.119	4.277	8.940	10.02
Negative likelihood ratio	0.513	0.510	0.281	0.172
ROC analysis AUC	0.767	0.767	0.885	0.928
Youden-index of cut-off	0.421	0.426	0.659	0.758

*CRH* corticotropic releasing hormone, *ITT* insulin tolerance test, *ROC* receiver operator curve, *AUC* area under the curve

### Early postoperative basal (nonstimulated) cortisol versus confirmation test

Mean postoperative basal cortisol concentrations in 156 patients were 321 nmol/L (range 9–909 nmol/L).

Sixteen patients had cortisol concentrations below 80 nmol/L. AI was confirmed during follow-up in all these patients (ITT *n* = 2, CRH test *n* = 3, ACTH test *n* = 1, basal cortisol *n* = 10). Postoperative basal cortisol was between 80 and 220 nmol/L in 26 patients, of whom 16 patients were diagnosed with AI during follow-up (ITT *n* = 3, CRH test *n* = 9, ACTH test *n* = 1, basal cortisol *n* = 3), and the other ten patients (38.5%) had normal adrenal function (ITT *n* = 7, CRH test *n* = 3) (Table 2, patients 7–16). Therefore, 24% of patients (10/42) with basal cortisol concentrations below 220 nmol/L had no AI during follow-up.

Postoperative basal cortisol was between 220 and 325 nmol/L in 39 patients. Of these, four patients (10%) were diagnosed with AI during follow-up (ITT *n* = 4, Table 3: patients 1–4). Thirty-five patients (90%) had normal adrenal function during follow-up (ITT *n* = 22, CRH test *n* = 13). Postoperative basal cortisol was above 325 nmol/L in 74 patients, and normal adrenal function was confirmed during follow-up in 72 patients (97%) (ITT *n* = 24, CRH test *n* = 23, basal cortisol *n* = 25). Two patients (3%) were diagnosed with AI during follow-up (CRH test *n* = 1, basal cortisol *n* = 1, Table 3: patients 5 and 6). One of these patients was tested during hypothyroidism, the other during an episode of SIADH. Both presented with complaints of AI, one with hyponatremia, the other with severe fatigue. Hence, only 6 out of 114 patients (5.3%) with early postoperative basal cortisol level above 220 were diagnosed with AI during follow-up. Performance indices of basal cortisol are presented in Table 4.

### Incidence of adrenal crisis

A total of six adrenal crises (grade 1 (*n* = 2), and grade 2 (*n* = 4)) occurred in four patients, during a mean follow-up of 4.2 years (range 0.7–8.6 years), and with a total of 656 patient-years of follow-up for the entire cohort. Three of these four patients had all been correctly identified after surgery as adrenal insufficient, and accordingly were put on hydrocortisone replacement. Adrenal crises occurred at 18, 28, 35, 54, 61, and 64 months, respectively, after surgery. One patient initially had been classified as false-negative based on the first postsurgical assessment, but this patient was correctly diagnosed with AI 6 months after surgery and was on hydrocortisone replacement thereafter (adrenal crisis occurred 54 months after surgery) (Table 3, patient 5).

## Discussion

In this study, the diagnostic performance of a single basal cortisol measurement proved to be superior to the CRH test for the assessment of postoperative adrenal function (see Table 4). We found that the performance of basal cortisol measurements was sufficient to guide the postoperative hydrocortisone replacement scheme, that such a strategy was safe, and did not result in any case of adrenal crises in potentially misclassified patients. However, there were discrepancies between the early postoperative basal cortisol and the confirmation test during follow-up.

There are no studies available that compared the CRH test and basal cortisol in the postoperative setting. In the (nonpostoperative) diagnostic setting, Dullaart et al. reported that basal cortisol was not inferior to the CRH test [16]. Schmidt et al. even advised against using the CRH test because of its low sensitivity [14]. In our study, both tests misclassified a small number of patients (false-negative test results: *n* = 6, 3.8%). Another two patients had false-negative results in the CRH test only (Table 3, patients 7 and 8). False-negative test results are of major concern in the assessment of adrenal function since untreated AI can potentially be life-threatening. As can be appreciated from Table 3, peak cortisol concentrations during dynamic confirmation tests were subnormal (between 328 and 424 nmol/L) in four of these eight patients, and very low (between 11 and 51 nmol/L) in the other four patients. The latter four patients are specifically intriguing, because of the large discrepancies between the early postoperative test results, and the test results during follow-up that can be explained by the following: first by the presence of untreated thyrotroph or somatotroph deficiency negatively affecting the cortisol response to stimulation. Thyroid hormone accelerates the endogenous clearance of cortisol [17], whereas growth hormone inhibits the conversion of cortisone to cortisol [18–21]. Consequently, the initiation or dose escalation of both thyroid hormone and growth hormone replacement can unmask impaired cortisol secretion. In agreement, two of these four patients had acquired secondary, and yet untreated, hypothyroidism after surgery (Table 3, patients 7 and 8), whereas the other two patients were already treated for preoperative secondary hypothyroidism (Table 3, patients 5 and 6). Three of the incorrectly classified patients had growth hormone deficiency. Another possible explanation is a subacute onset of AI in between the early postoperative evaluation and the second test, for example, due to late (pituitary) infarction. Finally, five of eight patients with false-negatives were tested during an (impending) episode of SIADH, or on irregular doses of desmopressin for DI. This may be explained by the co-stimulatory effect of vasopressin on ACTH secretion [22]. Of note, five of the eight patients reported severe fatigue prior to the diagnosis of AI

Vice versa, some discrepant test results in the group diagnosed with AI directly after surgery but with normal adrenal function during follow-up (*n* = 16) could be explained by late restoration of corticotroph function, or, although less likely, some suppression of corticotroph function due to the perioperative hydrocortisone replacement. In accordance, Pofi et al. recently demonstrated that restoration of adrenal function can occur even up to 12 months after surgery [23].

In the original assessment of the CRH test, the peak ACTH levels were incorporated. During this study, the peak ACTH levels altered none of the test results. An ROC analysis revealed an AUC of 0.673 (95%CI 0.551–796) versus all confirmation tests and of 0.639 (95%CI: 0.432–0.846) versus ITT alone. As the peak ACTH levels had no additional value for the test results, we omitted these data.

In a previous study, we evaluated the clinical applicability of postoperative CRH testing in our cohort treated from 1990 to 2009 [4]. The performance indices for the CRH test in that study were slightly lower than in the present study (sensitivity 67%, specificity 87%, PPV 69%, and NPV 86%). This can be explained, at least in part, by differing perioperative steroid replacement schemes, as dexamethasone was used in a majority of patients from 1990 to 2009 [4].

Studies have proposed different optimal cut-off values for early postoperative basal cortisol [14, 16]. The lower cut-off value obtained in this study is in agreement with already published data: 80 nmol/L (100 nmol/L with the cortisol I reagent) [12, 24, 25]. An exception is a study reported by Karaca et al., that suggested 129 nmol/L (165 nmol/L with cortisol I reagent) [10]. The two patients in our cohort with basal cortisol between 80 and 129 nmol/L had normal adrenal function during follow-up. The cut-off value for initiation of hydrocortisone replacement also differs between studies, with studies suggesting 234 [9], 312 [7] and 390 or between 299 and 390 nmol/L for clinically selected cases [8] (300, 400, 500, and between 370 and 500 nmol/L with cortisol I reagent, respectively). These discrepancies can be explained, at least in part, by differences between assays used, by different postoperative days of evaluation, by different perioperative glucocorticoid schemes, and by differences in treatment of other pituitary deficiencies. Therefore, centers should critically evaluate their own treatment and test results, and adjust cut-off values accordingly when implementing such a strategy.

A recent study by English et al. evaluated the accuracy of different tests for the assessment of postoperative adrenal function. They concluded that basal cortisol had a better diagnostic performance than a postoperative overnight metyrapone-suppression test. They also evaluated other tests and timing of the evaluation and concluded that a reliable formal assessment is only possible from 6 weeks postoperative onwards [7].

Limitations of this study are the retrospective evaluation and the fact that not all confirmatory tests during follow-up were done using the ITT, which is still considered the gold standard. As earlier stated, Schmidt et al. advised against using the CRH test because of its low specificity with the cutoff we used. However, the test results of all patients that underwent another stimulation test (CRH test or ACTH test) were concordant and also in agreement with clinical symptoms. Also, the reagent for the cortisol assay was changed during the study period. This did, however, not significantly affect outcomes as mean basal cortisol levels were not significantly different, 317 vs 300 nmol/L (*p* = 0.556) before and after the introduction of the new assay reagent. In addition, peak ACTH levels during CRH test, which assay was not altered during the study period, were also somewhat lower in the group analyzed after the cortisol assay change (mean ACTH 88 vs 70 ng/L).

**Fig. 3:**
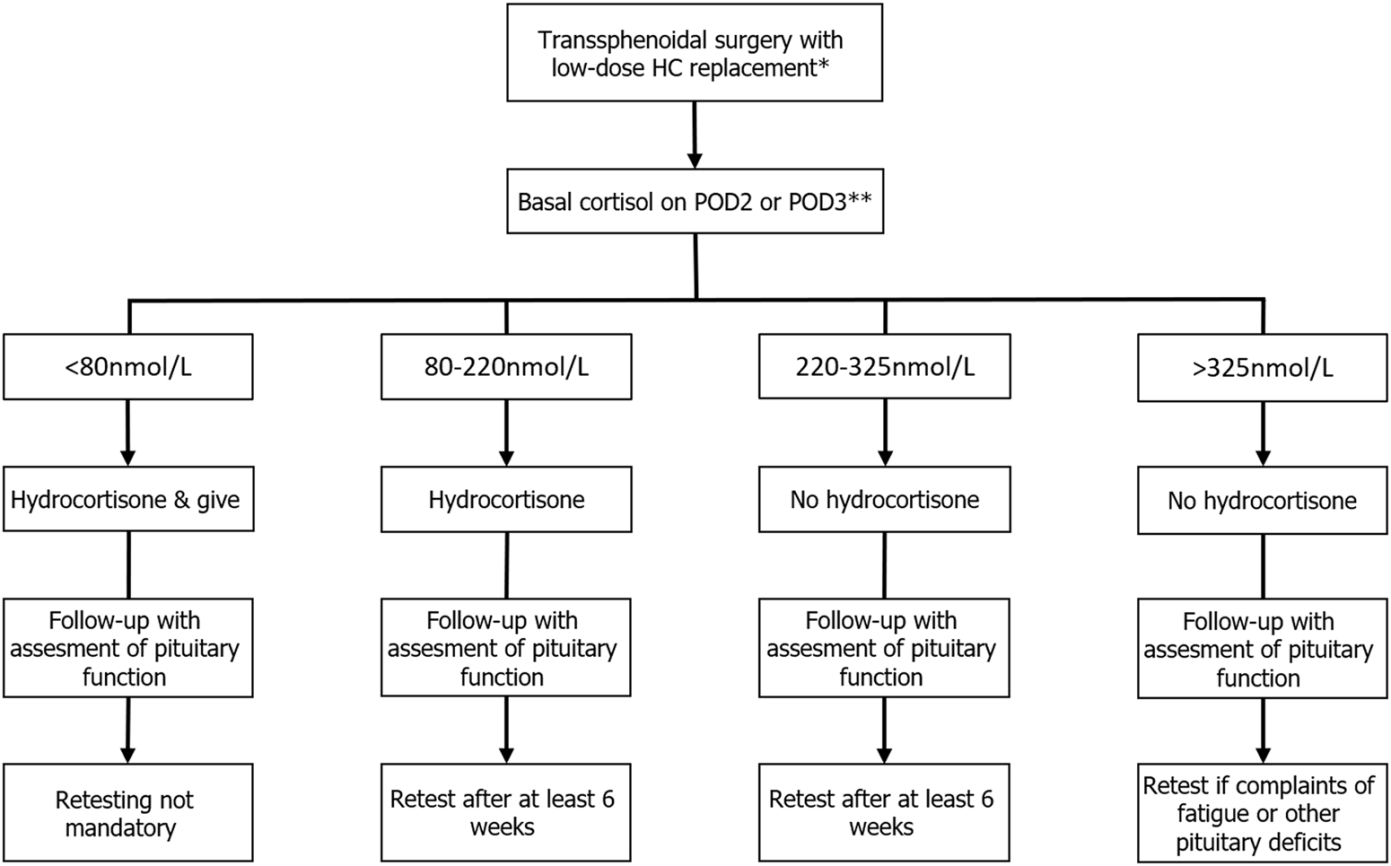
Proposed treatment regimen for postoperative testing. HC: hydrocortisone POD postoperative day, Asterisk indicates 50 mg of hydrocortisone/24 h on the day of operation, tapered to 10-5-5 mg of hydrocortisone on postoperative day 2, Double asterisk indicates If the patient has no diabetes insipidus, SIADH, CSF-leak or fever

In conclusion, the diagnostic performance of basal cortisol is sufficient to guide a postoperative hydrocortisone replacement scheme, and such a strategy is safe. The CRH test has no added value in this setting. Single, basal cortisol concentrations are easier to perform, more convenient for patients, and less costly. Consequently, we propose a new protocol for the assessment of adrenal function following pituitary tumor surgery (Fig. 3). Early morning basal cortisol samples should be obtained on the second or third day after surgery after withholding at least the evening and early morning dose of hydrocortisone. When basal cortisol is below 220 nmol/L hydrocortisone replacement should be continued. A formal assessment in those with postoperative basal cortisol between 80 and 325 nmol/L or symptoms suggestive of AI is advised at least 6 weeks after surgery. Reliability of testing can be optimized when testing is postponed in case of concomitant factors that can potentially affect ACTH and cortisol secretion and/or its bioavailability, i.e diabetes insipidus, SIADH, CSF-leakage, fever, and oral contraceptive use in the 6 weeks before testing [26].

## References

[1] Arraez MA (2013). Assessment of postoperative hypocortisolism after pituitary surgery: when and how?. World Neurosurg..

[2] Cerina V, Kruljac I, Radosevic JM, Kirigin LS, Stipic D, Pecina HI, Vrkljan M (2016). Diagnostic accuracy of perioperative measurement of basal anterior pituitary and target gland hormones in predicting adrenal insufficiency after pituitary surgery. Medicine.

[3] Klose M, Lange M, Kosteljanetz M, Poulsgaard L, Feldt-Rasmussen U (2005). Adrenocortical insufficiency after pituitary surgery: an audit of the reliability of the conventional short synacthen test. Clin. Endocrinol..

[4] Kokshoorn NE, Romijn JA, Roelfsema F, Rambach AH, Smit JW, Biermasz NR, Pereira AM (2012). The use of an early postoperative CRH test to assess adrenal function after transsphenoidal surgery for pituitary adenomas. Pituitary.

[5] Zada G, Tirosh A, Huang AP, Laws ER, Woodmansee WW (2013). The postoperative cortisol stress response following transsphenoidal pituitary surgery: a potential screening method for assessing preserved pituitary function. Pituitary.

[6] Courtney CH, McAllister AS, McCance DR, Bell PM, Hadden DR, Leslie H, Sheridan B, Atkinson AB (2000). Comparison of one week 0900 h serum cortisol, low and standard dose synacthen tests with a 4 to 6 week insulin hypoglycaemia test after pituitary surgery in assessing HPA axis. Clin. Endocrinol..

[7] English K, Inder WJ, Weedon Z, Dimeski G, Sorbello J, Russell AW, Duncan EL, Cuneo R (2017). Prospective evaluation of a week one overnight metyrapone test with subsequent dynamic assessments of hypothalamic-pituitary-adrenal axis function after pituitary surgery. Clin. Endocrinol..

[8] Hana V, JeZkova J, Kosak M, Krsek M, Marek J, Netuka D, Hill M, Hana V (2015). Prediction of adrenocortical insufficiency after pituitary adenoma surgery using postoperative basal cortisol levels. Physiological Res..

[9] Jayasena CN, Gadhvi KA, Gohel B, Martin NM, Mendoza N, Meeran K, Dhillo WS (2009). Day 5 morning serum cortisol predicts hypothalamic-pituitary-adrenal function after transsphenoidal surgery for pituitary tumors. Clin. Chem..

[10] Karaca Z, Tanriverdi F, Atmaca H, Gokce C, Elbuken G, Selcuklu A, Unluhizarci K, Kelestimur F (2010). Can basal cortisol measurement be an alternative to the insulin tolerance test in the assessment of the hypothalamic-pituitary-adrenal axis before and after pituitary surgery?. Eur. J. Endocrinol..

[11] McLaughlin N, Cohan P, Barnett P, Eisenberg A, Chaloner C, Kelly DF (2013). Early morning cortisol levels as predictors of short-term and long-term adrenal function after endonasal transsphenoidal surgery for pituitary adenomas and Rathke’s cleft cysts. World Neurosurg..

[12] Watts NB, Tindall GT (1988). Rapid assessment of corticotropin reserve after pituitary surgery. Jama.

[13] Hermus AR, Pieters GF, Pesman GJ, Benraad TJ, Smals AG, Kloppenborg PW (1987). CRH as a diagnostic and heuristic tool in hypothalamic-pituitary diseases hormone and metabolic research. Suppl. Ser..

[14] Schmidt IL, Lahner H, Mann K, Petersenn S (2003). Diagnosis of adrenal insufficiency: evaluation of the corticotropin-releasing hormone test and Basal serum cortisol in comparison to the insulin tolerance test in patients with hypothalamic-pituitary-adrenal disease. J. Clin. Endocrinol. Metabol..

[15] Allolio B (2015). Extensive expertise in endocrinology. Adrenal crisis.

[16] Dullaart RP, Pasterkamp SH, Beentjes JA, Sluiter WJ (1999). Evaluation of adrenal function in patients with hypothalamic and pituitary disorders: comparison of serum cortisol, urinary free cortisol and the human-corticotrophin releasing hormone test with the insulin tolerance test. Clin. Endocrinol..

[17] Persani L (2012). Clinical review: central hypothyroidism: pathogenic, diagnostic, and therapeutic challenges. J. Clin. Endocrinol. Metabol..

[18] Filipsson H, Johannsson G (2009). GH replacement in adults: interactions with other pituitary hormone deficiencies and replacement therapies. Eur. J. Endocrinol..

[19] Gelding SV, Taylor NF, Wood PJ, Noonan K, Weaver JU, Wood DF, Monson JP (1998). The effect of growth hormone replacement therapy on cortisol-cortisone interconversion in hypopituitary adults: evidence for growth hormone modulation of extrarenal 11 beta-hydroxysteroid dehydrogenase activity. Clin. Endocrinol..

[20] Giavoli C, Libe R, Corbetta S, Ferrante E, Lania A, Arosio M, Spada A, Beck-Peccoz P (2004). Effect of recombinant human growth hormone (GH) replacement on the hypothalamic-pituitary-adrenal axis in adult GH-deficient patients. J. Clin. Endocrinol. Metabol..

[21] Stewart PM, Toogood AA, Tomlinson JW (2001). Growth hormone, insulin-like growth factor-I and the cortisol-cortisone shuttle. Horm. Res..

[22] Salata RA, Jarrett DB, Verbalis JG, Robinson AG (1988). Vasopressin stimulation of adrenocorticotropin hormone (ACTH) in humans. In vivo bioassay of corticotropin-releasing factor (CRF) which provides evidence for CRF mediation of the diurnal rhythm of ACTH. J. Clin. Investig..

[23] Pofi Riccardo, Gunatilake Sonali, Macgregor Victoria, Shine Brian, Joseph Robin, Grossman Ashley B, Isidori Andrea M, Cudlip Simon, Jafar-Mohammadi Bahram, Tomlinson Jeremy W, Pal Aparna (2019). Recovery of the Hypothalamo-Pituitary-Adrenal Axis After Transsphenoidal Adenomectomy for Non–ACTH-Secreting Macroadenomas. The Journal of Clinical Endocrinology & Metabolism.

[24] Inder WJ, Hunt PJ (2002). Glucocorticoid replacement in pituitary surgery: guidelines for perioperative assessment and management. J. Clin. Endocrinol. Metabol..

[25] Marko NF, Hamrahian AH, Weil RJ (2010). Immediate postoperative cortisol levels accurately predict postoperative hypothalamic-pituitary-adrenal axis function after transsphenoidal surgery for pituitary tumors. Pituitary.

[26] Barel E, Abu-Shkara R, Colodner R, Masalha R, Mahagna L, Zemel OC, Cohen A (2018). Gonadal hormones modulate the HPA-axis and the SNS in response to psychosocial stress. J. Neurosci. Res..

